# Genenames.org: the HGNC and VGNC resources in 2021

**DOI:** 10.1093/nar/gkaa980

**Published:** 2020-11-05

**Authors:** Susan Tweedie, Bryony Braschi, Kristian Gray, Tamsin E M Jones, Ruth L Seal, Bethan Yates, Elspeth A Bruford

**Affiliations:** HUGO Gene Nomenclature Committee, European Molecular Biology Laboratory, European Bioinformatics Institute, Hinxton, Cambridgeshire CB10 1SD, UK; HUGO Gene Nomenclature Committee, European Molecular Biology Laboratory, European Bioinformatics Institute, Hinxton, Cambridgeshire CB10 1SD, UK; HUGO Gene Nomenclature Committee, European Molecular Biology Laboratory, European Bioinformatics Institute, Hinxton, Cambridgeshire CB10 1SD, UK; HUGO Gene Nomenclature Committee, European Molecular Biology Laboratory, European Bioinformatics Institute, Hinxton, Cambridgeshire CB10 1SD, UK; HUGO Gene Nomenclature Committee, European Molecular Biology Laboratory, European Bioinformatics Institute, Hinxton, Cambridgeshire CB10 1SD, UK; Department of Haematology, University of Cambridge School of Clinical Medicine, Cambridge, Cambridgeshire CB2 0AW, UK; HUGO Gene Nomenclature Committee, European Molecular Biology Laboratory, European Bioinformatics Institute, Hinxton, Cambridgeshire CB10 1SD, UK; HUGO Gene Nomenclature Committee, European Molecular Biology Laboratory, European Bioinformatics Institute, Hinxton, Cambridgeshire CB10 1SD, UK; Department of Haematology, University of Cambridge School of Clinical Medicine, Cambridge, Cambridgeshire CB2 0AW, UK

## Abstract

The HUGO Gene Nomenclature Committee (HGNC) based at EMBL’s European Bioinformatics Institute (EMBL-EBI) assigns unique symbols and names to human genes. There are over 42,000 approved gene symbols in our current database of which over 19 000 are for protein-coding genes. While we still update placeholder and problematic symbols, we are working towards stabilizing symbols where possible; over 2000 symbols for disease associated genes are now marked as stable in our symbol reports. All of our data is available at the HGNC website https://www.genenames.org. The Vertebrate Gene Nomenclature Committee (VGNC) was established to assign standardized nomenclature in line with human for vertebrate species lacking their own nomenclature committee. In addition to the previous VGNC core species of chimpanzee, cow, horse and dog, we now name genes in cat, macaque and pig. Gene groups have been added to VGNC and currently include two complex families: olfactory receptors (ORs) and cytochrome P450s (CYPs). In collaboration with specialists we have also named CYPs in species beyond our core set. All VGNC data is available at https://vertebrate.genenames.org/. This article provides an overview of our online data and resources, focusing on updates over the last two years.

## INTRODUCTION

The HUGO Gene Nomenclature Committee (HGNC) recently celebrated its 40th year as the global authority for assigning human gene symbols and names. HGNC works closely with authors, genome annotators, specialist advisors, expert resources and other nomenclature committees, to choose unique symbols and informative names. We name protein-coding genes, genes encoding RNA and pseudogenes; we do not name alleles and we no longer name phenotypes. We have recently published our updated naming guidelines (1), which reflect changes in policy in recent years and emphasise our aim to keep gene symbols stable where possible. We have also published a detailed set of guidelines specifically for naming RNA genes (2). All of our guidelines are summarised on our website, https://www.genenames.org.

In addition to an approved gene symbol and gene name, our core curated data also includes a unique HGNC ID, a locus type and a chromosomal location for each gene and, where appropriate, previous and/or alias nomenclature and membership of our manually curated gene groups (3). Mouse orthologs displayed in the human symbol report have also been largely manually curated by HGNC curators, and are tagged ‘curated’ - any without this tag have been downloaded from MGD (4). All other orthologs displayed in the human gene symbol reports are either imported from VGNC (see below), or in the case of rat downloaded from RGD (5). We also provide orthology predictions for 19 species via our HCOP tool from a separate tab on the gene report. HCOP aggregates and displays the orthology assertions from 15 different resources (6).

HGNC nomenclature data is displayed by many external resources including NCBI Gene (7), Ensembl (8), UniProt (9), GeneCards (10), RNAcentral (11) and the UCSC genome browser (12), as well as resources focused on human disease and phenotypes such as Decipher (13), OMIM (14), ClinGen (15), ClinVar (16) and GeneTests (17). HGNC is a recommended resource on FAIRsharing (18) and became an Elixir UK service node in 2020 (19).

Our younger sister project, the Vertebrate Gene Nomenclature committee, is now in its 5th year of naming genes in selected vertebrate species that do not have their own dedicated nomenclature committee (20). Nomenclature assignment in VGNC is semi-automated, with automatic transfer of human nomenclature to high confidence 1:1 orthologs in other species (i.e. where there is full agreement across the orthology calls made by Panther (21), NCBI Gene (7), OMA (22) and Ensembl (8)) and manual assignment of nomenclature to the remaining genes.

Both HGNC and VGNC databases are freely available to all via the web without the need to register or login and are accessible and legible on phone and tablet screens. All data is available for download in both txt and JSON format.

We describe the changes made to the HGNC and VGNC resources since our last report in 2019 (23).

## HGNC DATA

### New genes named

We named 1414 new human genes in the last two years; the majority of these were non-coding RNA encoding genes (787) and pseudogenes (587). Numbers of newly named protein-coding genes remain comparatively low (28 in the last 2 years) because our protein-coding dataset is relatively complete, but novel approaches to detecting protein-coding genes continue to yield new gene models; for example, we worked closely with GENCODE annotators to name novel protein-coding genes identified using PhyloCSF (24). For non-coding RNA genes, our long non-coding RNA (lncRNA) dataset continues to grow the fastest with 704 new lncRNA symbols since September 2018. A small proportion of these genes were named after information from a publication such as *WAKMAR2* (25) and *LERFS* (26), while 284 lncRNA genes were named based on being antisense to a protein-coding gene, 269 as intergenic, and 99 as divergent transcripts which share a bidirectional promoter with a protein-coding gene (2). We have recently updated the format of our lncRNA gene group page (https://www.genenames.org/data/genegroup/#!/group/788) to support quick viewing and counting of lncRNA genes by symbol format. Since September 2018, we have also named 60 microRNAs in collaboration with miRBase (27), 15 transfer RNAs in collaboration with GtRNAdb (28), and 8 variant small nuclear RNAs following consultation with our specialist advisor Dawn O’Reilly.

### Phenotypes symbols withdrawn

Overall the number of approved symbols has increased since our last update publication, but only by ∼700 to give a current total of 42 185—this reduced increase is largely due to the fact that all 569 approved entries which had the locus type ‘phenotype’ have now been formally withdrawn. As OMIM has taken over the cataloguing and assignment of human phenotype symbols, and our set of ‘phenotype’ symbols was not complete, nor being regularly updated, we made the decision to withdraw all of these entries. The withdrawn entries are still searchable from our website, and have retained key information in the symbol report - such as the approved symbol and name, chromosomal location and HGNC ID, as well as linked publications—but they are no longer being updated. In line with all of our withdrawn entries they are now marked with a red triangle to remind users the entry is no longer approved. Note that all requests for new phenotype symbols should be directed to OMIM (14).

### Stabilization of gene symbols

**Figure 1.**
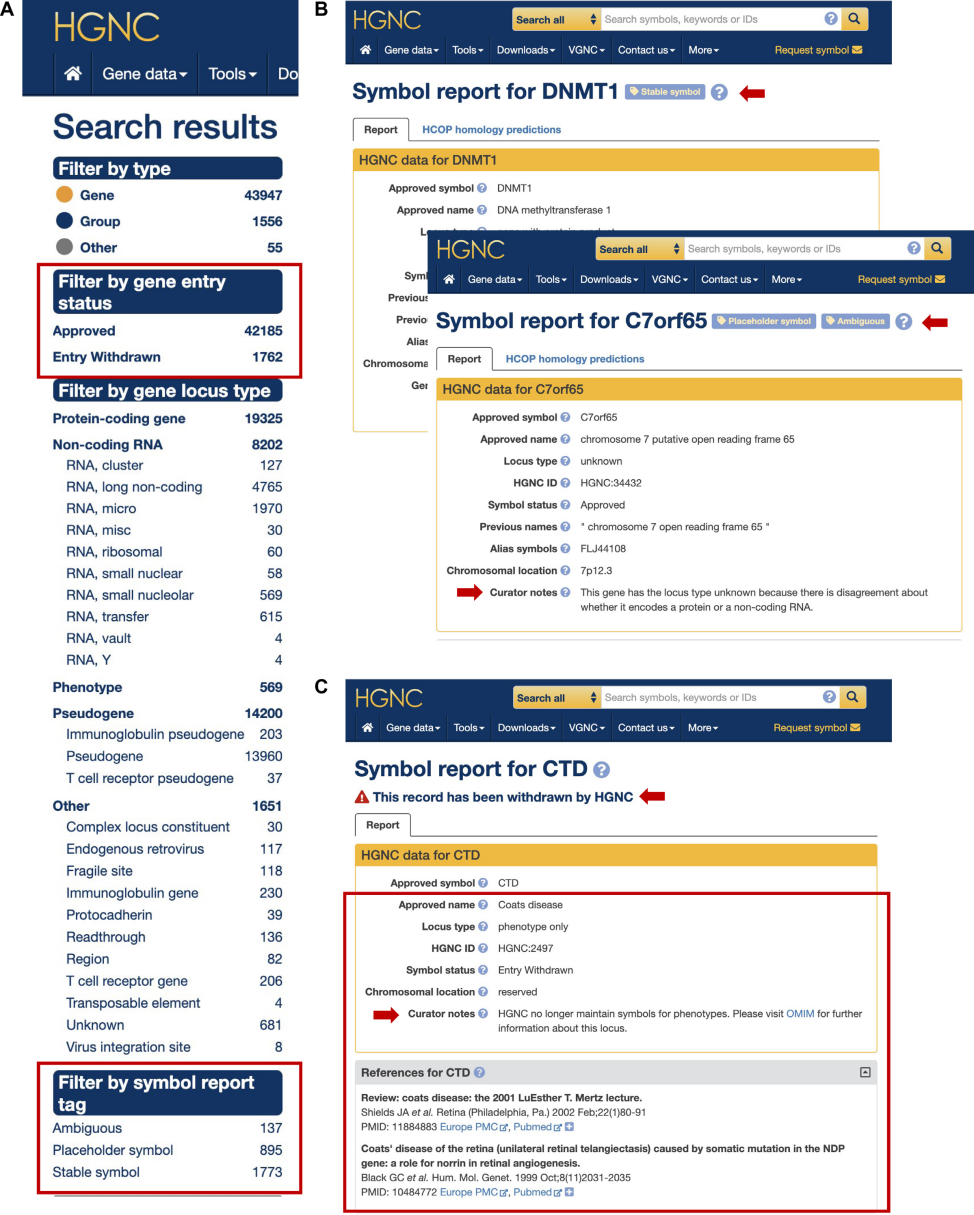
Screenshots showing new HGNC website features. (**A**) Screenshot of the left hand side of our ‘query all data’ page, with counts by data type. New facets for filtering data are boxed. (**B**) Examples of the top section of HGNC symbol reports displaying new symbol tags and curator notes (both indicated with arrows). (**C**) An example symbol report for a withdrawn entry with a new warning triangle (arrowed). Data previously absent from our symbol reports for withdrawn entries, in this case including a new curator comment (arrowed), is boxed.

Changes to gene symbols can be disruptive and cause confusion, and in the case of genes associated with disease could even result in an incorrect diagnosis and treatment. Consequently, our symbol updates are now limited and occur only in scenarios where the benefits of change clearly outweigh the disadvantages (1). In an effort to provide additional stability for genes associated with disease, we have been reviewing their nomenclature and marking them with the tag ‘stable symbol’ when we are confident that future symbol changes are extremely unlikely. The review process considers a number of factors, including usage of the current approved symbol relative to alternative/alias symbols, whether the current symbol is misleading in any way or potentially offensive or upsetting to a patient with a disorder related to the gene, or whether it is causing significant problems for literature searching or data processing. To date over 2000 genes have been marked as being a stable gene symbol—these are indicated by our new ‘stable symbol’ luggage tag on our gene reports (see HGNC Website section below and Figure 1A, B).

### Gene symbol updates

Although we now strive for symbol stability, there are still some cases where updates can be justified. When we identify a symbol that we think should change, we consult as widely as practical with users of the symbol to gauge if the change is deemed necessary and to agree a suitable alternative.

For example, we have updated the symbols for a set of genes that were being auto-converted to dates when entered in Microsoft Excel. It had become clear that this very widespread issue could not be solved by advising users to change their settings in Excel so, after consultation with the research community publishing on these genes, we updated their nomenclature. All MARCH# symbols were changed to MARCHF#, all MARC# symbols to MTARC#, all SEPT*#* symbols to SEPTIN# and the symbol *DEC1* became *DELEC1*.

We also felt compelled to change a small number of symbols that could be perceived as pejorative, particularly when associated with a phenotype, e.g. *DOPEY1* and *DOPEY2* have been updated to *DOP1A* (DOP1 leucine zipper like protein A) and *DOP1B* (DOP1 leucine zipper like protein B).

We consider updating gene symbols where the current symbol and name could be misleading. An example is *ISPD* (isoprenoid synthase domain containing), which was originally named based on the presence of an isoprenoid domain. This domain is derived from an ancestral gene that, in plants, protozoa, and some bacteria is involved in a non-mevalonate/MEP pathway of isoprenoid synthesis (29), but the pathway is not present in humans so we took the decision to update the nomenclature of *ISPD* to *CRPPA* (CDP-l-ribitol pyrophosphorylase A) thus giving the gene a functionally informative name that is not misleading.

We have continued to update ‘placeholder’ symbols (those with C#orf#, FAM and KIAA roots) when possible, and in total 69 have been updated since our last report. This leaves 534 placeholder symbols approved for protein-coding genes that we remain keen to update; these are now marked with a ‘Placeholder’ luggage tag at the top of the Symbol Report (Figure 1B). Researchers with information about the function of these genes are encouraged to contact us to discuss new symbol suggestions prior to publication.

C#orf# symbols are assigned to predicted genes designated by the chromosome of origin, the letters ‘orf’ for open reading frame and an iterative number. We have updated the nomenclature for 31 protein-coding C#orf#s since our last report. Examples include changes based on enzyme activity e.g. *C15orf41* is now *CDIN1* (CDAN1 interacting nuclease 1), updates based on the presence of shared domains or motifs in gene protein products e.g. *C1orf123* is now *CZIB* (CXXC motif containing zinc binding protein), and updates based on gene products being published as subunits in complexes e.g. *C7orf43* changing to *TRAPPC14* (trafficking protein particle complex 14).

KIAA# symbols are approved for genes identified by the Kazusa cDNA sequencing project when no other information is known about the gene. We have updated the symbols for 10 KIAA#s including *KIAA0355* to *GARRE1* (granule associated Rac and RHOG effector 1) and *KIAA1551* to *RESF1* (retroelement silencing factor 1).

The FAM# root is used to group together a set of genes that are related based on sequence similarity that cannot be described by function or conserved domains. We have updated 13 sets of FAM# symbols since our last report, consisting of 28 individual genes in total. Examples include updating *FAM84A* and *FAM84B* to *LRATD1* and *LRATD2* (LRAT domain containing 1 and 2) and, based on overwhelming usage of an alias in the literature, the FAM129 family were renamed using the root NIBAN *(*niban apoptosis regulator).

### New curator notes added

We have added a new field to the core data section of our Symbol Reports entitled ‘Curator Notes’ (Figure 1B and C). This field allows us to explain particular aspects of the symbol, name, status or locus type in free text. In many cases, these notes have a controlled vocabulary and are applicable to multiple genes. For example, all 206 pseudogenes that have been named after a protein-coding ortholog from another species (such as *PFN5P* which has no human parent gene but is named relative to the functional mouse gene *Pfn5*) now include the curator note: ‘This pseudogene has been named based on its functional ortholog in another species’. Our recently-withdrawn phenotype entries, mentioned above, contain the note ‘HGNC no longer maintain symbols for phenotypes. Please visit OMIM for further information about this locus’ and although the text is the same in all instances, the link to OMIM is specific to each gene (Figure 1C). Some curator notes apply to only one gene, such as the curator note ‘An alternative protein nomenclature proposed in Groll *et al.* calls the *PSMB10* gene product the beta 2i subunit of the 20S proteasome’ which is specific to the *PSMB10* Symbol Report. It is possible to search genenames.org for curator note text by selecting the option ‘genes’ and prefixing the quoted search text with the field tag ‘curator_notes:’ e.g. curator_notes:’OMIM’ returns the 352 withdrawn phenotype entries with an OMIM link.

### Gene groups

In the last two years we have added just over 300 new manually curated gene groups, bringing the total number of groups to 1556 (as of September 2020). HGNC gene groups are based on a variety of shared characteristics including homology, domains, membership of a complex and genomic location; many of the groups relate to the basis of our approved nomenclature for the genes within the group and often provide groupings that are not available in other resources. A group will often share a common root symbol (indicated on the group report) but there are many examples where specific members have been named differently and it is not appropriate to change a well used symbol for the sake of consistency. Hence our group reports make it easy to identify gene groups even though they may have disparate symbols. Examples of recently added gene groups include: ‘Caveolins’ (CAV), a group of three paralogs that share the CAV# root symbol; ‘Radical S-adenosylmethionine domain containing’ (RSAD) which includes *RSAD1* and *RSAD2* with the remaining seven members having different root symbols; ‘Dolichyl-phosphate mannosyltransferase subunits’ (DPM)’ groups the catalytic (*DPM1*) and regulatory subunits (*DPM2* and *DPM3*) of this enzyme; ‘Mitochondrial genome’ and a series of subgroups including ‘Mitochondrially encoded regions’ (non-genic regions with approved symbols).

## HGNC WEBSITE UPDATES

### Improved display of withdrawn entries

We have improved the way we display and support searching of our withdrawn entries. Previously, the gene symbols of withdrawn entries were displayed with the term ‘∼withdrawn’ appended after the symbol, which meant that in order to find withdrawn entries on genenames.org it was necessary to add a wildcard when using our ‘Search all’ function, i.e. the search term BLYM* was necessary to return BLYM∼withdrawn. It is now possible to find the correct withdrawn entry simply by searching with the former approved symbol.

Previously we removed all data from the symbol report, other than the gene symbol itself – the locus type and gene name fields were displayed as ‘Entry Withdrawn’ only. We now have ‘Entry Withdrawn’ as the symbol status and display more of the original data for the entry including the gene name, locus type and references (Figure 1C). A new curator note can also be added to explain why the entry has been withdrawn. We have also added a red warning triangle followed by the text ‘This record has been withdrawn by HGNC’ to the top of each relevant Symbol Report to alert users that these are no longer approved gene symbols (Figure 1C).

To allow filtering of withdrawn entries, we have added the facet ‘Filter by gene entry status’ to our search function (Figure 1A). If search results contain withdrawn entries, this filter gives the option ‘Entry withdrawn’ in addition to ‘Approved’, e.g. searching with the root symbol CYP* shows that there are three withdrawn entries, in addition to the 131 approved entries.

### Addition of symbol report tags

We have introduced luggage style tags to our symbol reports (Figure 1B)—currently there are 3 types of tag:

- ‘*Placeholder symbol’—*this is added to the three main groups of symbols that we consider temporary: C#orf#s, KIAAs and FAMs. We welcome symbol update requests for these genes.
- *‘Stable symbol’—*used to indicate symbols that have been reviewed and are considered very unlikely to change.
- ‘*Ambiguous*’—used to indicate when the locus type is uncertain; a curator note details the nature of the uncertainty.

We have added the facet ‘Filter by symbol report tag’ to our search, so that it is possible to find genes associated with each type of symbol tag (Figure 1A).

### New links to other resources

We now link from our symbol reports to the human gene pages in the Alliance of Genome Resources Portal (30), which provides a convenient route to relevant information in the main model organism databases and the Gene Ontology. We have also added links to the Monarch Initiative, a multi-species database of integrated genetic, phenotypic, and disease data (31).

### New downloadable data files

The HGNC website updates daily rather than producing periodic releases. While this allows us to make data available quickly it also makes it more difficult for users to return to a specific HGNC data set and compare our datasets over time. To address this we now archive the complete HGNC dataset file (in both tab separated and JSON formats) each month and each quarter. These files can be found in the archive section of our FTP site (links are provided from our Statistics & download files page https://www.genenames.org/download/statistics-and-files/). We recommend using one of these dated files if you intend to use our data in a published study, so that other researchers have access to a traceable dataset. We have also created a file that contains all of our withdrawn entries and their associated public data, which can again be found linked from our statistics and downloads page.

In addition to these files, we also provide a custom download tool which includes the option to search ‘Date approved’, ‘Date symbol changed’ and ‘Date name changed’ to identify newly approved genes or track the latest nomenclature changes.

### New HGNC/VGNC blog

The genenames blog (https://blog.genenames.org/) was launched in April 2019 and can be accessed via links on the HGNC and VGNC homepages or by clicking the ‘blog icon’ found in our website footers. The blog provides a place to publish our newsletters, but also hosts discussion posts highlighting nomenclature issues and explaining new developments within our projects. We also publish ‘how-to’ guides and guest posts from our Scientific Advisory Board members and collaborators. New posts are announced via our Twitter account, @genenames.

## VGNC DATA AND WEBSITE UPDATES

### New core species added

The VGNC is now approving nomenclature in seven vertebrate species: chimpanzee, horse, cow, dog, macaque, cat, and pig. The latter three species were added to the VGNC database in May 2019 (macaque and cat) and January 2020 (pig). As of September 2020, the VGNC has approved symbols and names for 101 750 genes, with over 12 000 genes named in each of the core species, i.e. species for which we aim to name all protein-coding genes. While our focus is largely on approving nomenclature for protein-coding genes at present, we have also approved nomenclature for over 500 pseudogenes.

We collaborate with external specialists to assign nomenclature to members of large gene families, namely the olfactory receptors (ORs) and the cytochrome P450s (CYPs). In April 2020, we published our nomenclature system for the OR family in vertebrates (32) and manual curation of these genes is in progress in our core VGNC species. For members of the CYP family we include data from additional species in addition to the core VGNC species. We currently have over 900 cytochrome P450 genes approved across 17 additional primate species, and plan to continue approving cytochrome P450 genes in additional species in collaboration with our external specialists for this gene family.

A list of all VGNC species and the number of genes named in each is available at https://vertebrate.genenames.org/about/species-list/. All VGNC data is available via the Downloads tab on the VGNC site, and we recently added a dropdown menu to the top of the Statistics and Downloads page that allows selection of data for a single species.

### VGNC website redesign

**Figure 2.**
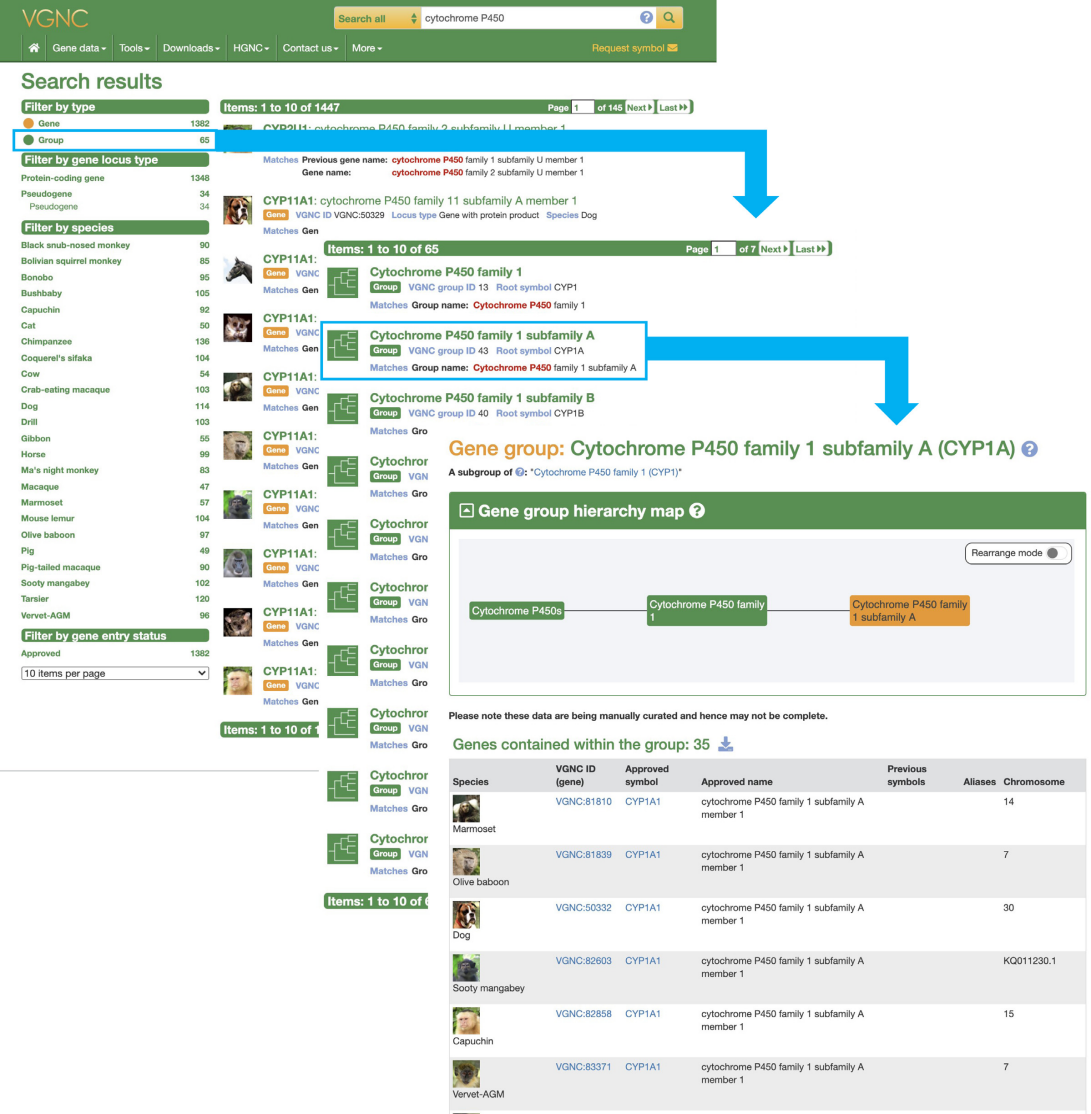
A series of screenshots illustrating the new VGNC Gene groups. The left hand panel shows a search result, the middle panel the result of filtering for ‘Group’ hits, and the right hand panel the top section of the selected Gene group report. Clicking on the boxed features allows navigation between the pages (as indicated by the arrows).

In late 2019, we released a new version of our VGNC website https://vertebrate.genenames.org. While not appearing radically different to our users this redesign brought the VGNC website in line with the current HGNC site, both in terms of the look and feel of the site and the underlying web technologies used, which will allow us to easily share any new web features we develop across both sites. A major addition to the new VGNC website is the gene group display which mirrors the gene group display found on the HGNC website (Figure 2). Currently much of the data found within the VGNC gene groups relates to cytochrome P450 genes and olfactory receptor genes contributed by our gene family experts. As VGNC gene groups are not species specific they provide a convenient way to display differences within a specific gene family across a taxonomic divide. There is a gene group specific search on the VGNC website and gene group data is downloadable both as a complete dataset or by family and subfamily.

## FUTURE PLANS

### HGNC

We will continue reviewing nomenclature of disease associated genes and increase the number of stabilised symbols. We will also continue to systematically approve nomenclature for all newly identified human protein-coding genes, as well as consistently annotated and published non-coding RNA genes and pseudogenes. We will continue to update temporary placeholder symbols to more appropriate function-based nomenclature whenever possible. We will also continue to create new gene group pages, particularly when they relate to the nomenclature of human genes.

We plan to expand and improve the functionality of our search by adding autocompleted query suggestions as the user types into the search input box. The improved search will also allow the user to search with the beginning of a symbol without having to use a wildcard. It will also allow a singular query term (e.g. kinase) to match plurals (e.g. kinases) and vice versa.

We plan to add a new InterMine service (33) for downloading our data, and to bring us in line with the multiple model organism databases which also have InterMine services. We will also implement a new containerised REST API built upon MongoDB and Nginx using the restify framework and OpenAPI/Swagger.

### VGNC

We will continue to approve protein-coding genes in our seven core VGNC species using a combination of automated and manual approaches. We may add additional species to our pipeline depending on community demand and the quality of the genome build.

We will continue to work with specialists to improve the coverage in our VGNC existing gene groups and add new gene groups. We will also add the option to filter gene group displays to show selected species only.

We plan to implement a new containerised REST API and InterMine service (33) in line with those being developed for HGNC.
